# Immune responses to azacytidine in animal models of inflammatory disorders: a systematic review

**DOI:** 10.1186/s12967-020-02615-2

**Published:** 2021-01-06

**Authors:** Sija Landman, Chiel van der Horst, Piet E. J. van Erp, Irma Joosten, Rob de Vries, Hans J. P. M. Koenen

**Affiliations:** 1 Department of Laboratory Medicine-Laboratory Medical Immunology, Radboud Institute for Molecular Life Sciences, Radboudumc, Nijmegen, The Netherlands; 2 Department of Dermatology, Radboudumc, Nijmegen, The Netherlands; 3 Systematic Review Centre for Laboratory Animal Experimentation (SYRCLE), Department for Health Evidence, Radboud Institute for Health Sciences, Radboudumc, Nijmegen, The Netherlands

**Keywords:** Azacytidine, Animal models, Inflammatory disorder, Systematic review

## Abstract

Inflammatory disorders like diabetes, systemic lupus erythematodes, inflammatory lung diseases, rheumatoid arthritis and multiple sclerosis, but also rejection of transplanted organs and GvHD, form a major burden of disease. Current classes of immune suppressive drugs to treat these disorders are never curative and side effects are common. Therefore there is a need for new drugs with improved and more targeted modes of action. Potential candidates are the DNA methyl transferase inhibitor 5-azacytidine (Aza) and its derivative 5-aza 2′deoxycitidine (DAC). Aza and DAC have been tested in several pre-clinical in vivo studies. In order to obtain an overview of disorders for which Aza and/or DAC can be a potential treatment, and to find out where information is lacking, we systematically reviewed pre-clinical animal studies assessing Aza or DAC as a potential therapy for distinct inflammatory disorders. Also, study quality and risk of bias was systematically assessed. In the 35 identified studies, we show that both Aza and DAC do not only seem to be able to alleviate a number of inflammatory disorders, but also prevent solid organ rejection and GvHD in in vivo pre-clinical animal models. Aza/DAC are known to upregulate FOXP3, a master transcription factor for Treg, in vitro*.* Seventeen studies described the effect on Treg, of which 16 studies showed an increase in Treg. Increasing Treg therefore seems to be a common mechanism in preventing inflammatory disorders by Aza/DAC. We also found, however, that many essential methodological details were poorly reported leading to an unclear risk of bias. Therefore, reported effects might be an overestimation of the true effect.

## Background

Epigenetic mechanisms such as (de)methylation of DNA/RNA are important for the development and function of the immune system. A failure to maintain epigenetic homeostasis may lead to aberrant gene expression, and contribute to immune dysfunction including the development of autoimmunity and autoinflammatory disorders [1]. Epigenetic modifiers are studied as potent drugs to regulate the immune system [2]. A drug with much potential is 5-azacytidine (Aza, Vidaza) and its derivative 5-Aza-2′-deoxycytidine (DAC, Decitabine)[2]. Aza and DAC are cytidine analogs which are incorporated into DNA/RNA and DNA respectively [3]. Incorporation of Aza/DAC leads to inactivation of DNA methyltransferases (DNMT), thereby leading to hypomethylation of DNA. Genes are silenced by hypermethylation, therefore demethylation leads to increased gene expression [4]. In high concentrations, Aza and DAC are cytotoxic to abnormal hematopoietic cells [3].

Currently, Aza and DAC are used as a treatment for hematological malignancies, including myelodysplastic syndrome (MDS) and acute myeloid leukemia (AML) when stem cell transplantation is not an option, mainly in elderly patients [5, 6]. The anticancer effect is thought to depend on promoting anti-tumor genes by hypomethylation and direct cytotoxic effects to abnormal hematopoietic cells [7, 8]. In MDS and AML patients, upon Aza treatment, changes in CD4 + , CD8 + and Treg numbers were found [9].

Treg are anti-inflammatory cells and important for the homeostasis of the immune system. Stability of Treg is controlled by stable expression of the transcription factor Forkhead Box P3 (FOXP3) [10, 11]. In vitro*,* Tconv and Treg were shown to further upregulate FOXP3 upon cell stimulation in the presence of Aza/DAC, which is explained by hypomethylation of the Treg-specific demethylated region (TSDR) within the FOXP3 gene [12, 13]. Immune-disturbances of Treg in auto-immune/auto-inflammatory disorders such as diabetes, psoriasis and in graft versus host disease (GvHD) cause a shift to a pro-inflammatory response [14]. Upregulating Treg or promoting Treg stability via epigenetic modification is therefore a promising new treatment option [2].

Because of the promising in vitro results, Aza and DAC are also being studied in animal models of auto-immune and auto-inflammatory disorders. These in vivo studies vary widely in disease-, drug regimen- and cells studied as outcome. Combining and comparing all different in vivo animal studies may lead to new insights regarding the use of Aza/DAC in clinical disease. It may also lead to a reduction and refinement of animals used, because of design of improved study protocols. We therefore systematically reviewed the existing literature on Aza and DAC used as treatment in animal models of inflammatory disorders to study its effect on the immune system. This systematic review contains: a complete overview of animal studies on this topic based on a comprehensive search strategy, an overview of clinical outcomes of all selected studies, summarized evidence for the effect of treatment of inflammatory disorders with Aza and DAC on immune outcomes, and an assessment of the risk of bias and reporting quality of the selected animal studies.

## Methods

### Review protocol

This systematic review investigates the effect of azacytidine (Aza) and decitabine (DAC) on immune-related outcomes in animal models for inflammatory disorders. The inclusion and exclusion criteria and method of analysis were specified in advance and documented in a protocol registered on PROSPERO (https://www.crd.york.ac.uk/prospero) with registration number CRD42018088017 on 26-02-2018. The protocol is based on the protocol format from SYRCLE (https://www.syrcle.nl) [15].

### Search strategy and selection of relevant studies

A comprehensive search string to identify studies assessing the use of Aza or DAC as a treatment for inflammatory disorders in an animal model was generated. The databases Pubmed and Embase were used to find all primary studies (date last search: 27-09-2019). The search strategy consisted of three search components: Aza or DAC, inflammatory disorders and animal studies. The search strategies can be found in Additional file 1: Table S1. The search filters for animal studies for Embase and Pubmed were used from SYRCLE [16, 17].

The studies found by the search strategy of both Pubmed and Embase were combined and cleared of duplications. Papers were screened by two independent investigators (SL and CvdH) with the use of Early Review Organizing Software (EROS; Institute of Clinical Effectiveness and Health Policy, Buenos Aires, Argentina). Studies were included when Aza or DAC was used in an animal model for an inflammatory disease with immune outcomes. Inflammatory disorders were defined as non-infectious inflammation including auto-immune and auto-inflammatory disorders, organ rejection after transplantation and GvHD. Studies were excluded when: Aza or DAC was not used, no inflammatory disease was studied, the study did not contain an animal model, the study was not a primary study (for example a review or conference abstract) or the study had no immune outcomes. Disorders were excluded when bacterial or viral infections were used without the purpose of inducing an inflammatory disease. Also cancer, myocardial infarction, fibrosis and sickle cell anemia were excluded because we did not consider them as an inflammatory disease even though they have inflammatory components. No restrictions on language were used both during the search and selection.

The first selection of relevant studies was done based on the abstract and title by two independent reviewers (SL and CvdH). In case of doubt whether a study should be included the whole article was evaluated. In case of differences between both investigators a third investigator was consulted (PvE). After the first selection, a second selection was done based on the whole article. The same inclusion and exclusion criteria were used and in case of differences a third investigator was consulted.

### Data extraction and synthesis

The following characteristic were extracted from the studies by two independent investigators (SL and CvdH): study design (control group used, experimental group used, group size), animal model characteristics (animal species, sex), disease model used (modeled disease, type of induction of the disease), intervention characteristics (Aza or DAC used, dose of drug used, dose regimen used, route of administration) and clinical outcome. In case of discrepancies, the investigators discussed the study and found agreement.

Induction of Treg upon Aza/DAC treatment is a common hypothesis for the immune modulatory effect these drugs. Therefore we summarized the effect of Aza/DAC treatment on FOXP3/Treg cells. Because the number of studies per inflammatory disease was too low and the heterogeneity in characteristics between the studies was too high, we decided not to combine the outcome data in a meta-analysis. Instead, we created a table displaying the direction of effect as reported by the authors per immune outcome. An overview was made of significant changes in the immune cell populations of leukocytes (CD45), granulocytes, lymphocytes, macrophages, T cells (CD3), T-helper cells (CD4), cytotoxic T cells (CD8) and B cells (B220). We also made an overview of the commonly measured cytokines IL-1β, IL-4, IL-5, IL-6, IL-10, IL-12, IL-13, IL-17, TNFα, TGFβ and IFNγ.

### Quality assessment

SYRCLE Risk of bias (RoB) tool was used to assess the risk of bias in the included studies [18]. A ‘yes’ score indicates low risk of bias; a ‘no’ score indicates a high risk of bias; and a ‘?’ score indicates unclear risk of bias. Because the reporting of experimental details on animals, methods and materials tends to be very poor, we added a few items on the quality of reporting: reporting of: any blinding, any randomization, any housing conditions, ethical approval and any conflict of interest. Two independent reviewers (SL and CvdH) assessed the RoB and reporting quality in all included studies.

## Results

### Study selection

**Fig. 1:**
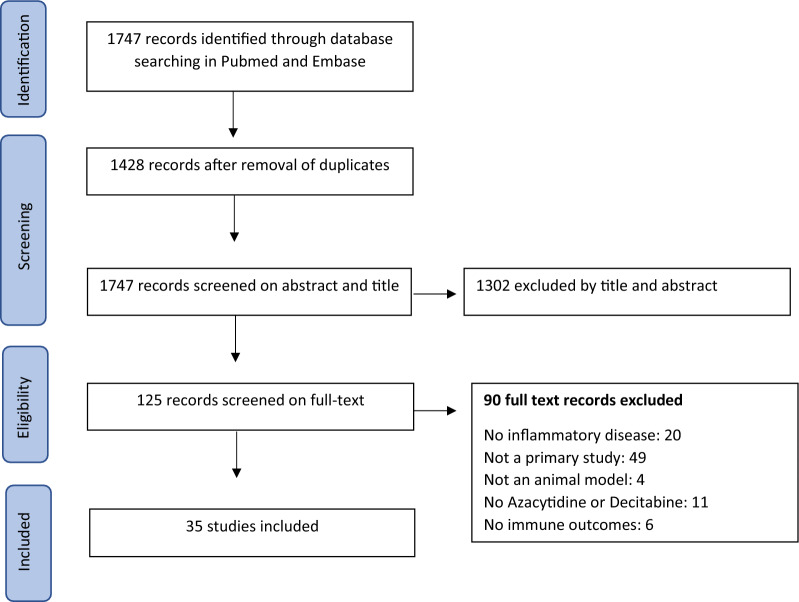
PRISMA chart of study selection process. Duplicate references are references that are mentioned in multiple medical databases (e.g. same references in Embase and Pubmed) or studies published in duplicate (e.g. same study in multiple journals)

Searching Pubmed and Embase for references regarding Aza and DAC on immune-related outcomes in animal models for inflammatory disorders resulted in 1747 references. Removal of duplicates left 1427 papers for screening based on abstract and title. 125 studies passed the abstract and title screening, of which 35 studies were left after full text screening. Figure 1 shows the PRISMA chart of the study selection process.

### Study characteristics

In the 35 studies included, Aza was used 18 times and DAC was used in 12 of the cases. In 5 studies, both Aza and DAC were used. Most studies described the dose as grams per kg. Doses of Aza used varied from 0.15 to 25 mg/kg. Doses of studies describing the dose in μg/dose varied from 10 to 100 μg. It was unfortunately not possible to convert this dose to mg/kg. One study used nanolipogels to specifically target CD4 + or CD8 + T cells. This study used a dose of 5 μg/dose. Doses of DAC used varied from 0.1 to 20 mg/kg. One study treated cells ex vivo with 0.5 μM DAC before infusion of cells. Drugs were administered intraperitoneally (28 times), intravenously (2 times) or subcutaneously (3 times). In three studies, the route of administration was not well described. Thirty-one studies used mice, 1 used rats, 2 used both mice and rats, and 1 study used chickens. Sixteen studies used male animals, while 6 used female animals; use of both sexes was reported in 6 cases, while in 6 studies sex was not mentioned. One study mentioned that they used cells from male mice for in vitro studies, but it was unclear whether this was also the case for the in vivo studies. Study characteristics are shown in Table 1.

**Table 1:** Characteristics of the included studies

Author first, last, year, journal	Species	Sex	Type of induction of disease	Aza or DAC	n/group	Dose	Dose regimen (frequency and timing)	Route of administration	Clinical outcome
Graft versus host disease (GvHD)									
Paluska Cihak 1982 Immunobiology*	Mice	M+F	Allogeneic splenocyte injection	Aza and DAC	8–17	6 or 20 mg/kg	24 h after splenocyte injection	IP	Decreased GvHD, depending on dosing
Sula, Cihak, 1987, Czech Med	Mice	Unknown	Allogeneic splenocyte injection	Aza and DAC	5–10	5-25 mg/kg	Day -1, 0, 1, or 2 or multiple days	IP	Decreased GvHD, depending on dosing
Sánchez-Abarca, Pérez-simon, 2010, the american society of hematology	Mice	M+F	BM transplantation + allogeneic splenocyte injection	Aza	5	1 mg/kg	after 60 and 84 h	IV	Decreased GvHD, increased survival
Choi, Dipersio, 2010, the american society of hematology	Mice	Unknown	BM transplantation + allogeneic T cell injection	Aza and DAC	2–25	DAC: 1mg/kg , Aza: 2 mg/kg	Day 4, 6, 8, 10 after T cells	SC or IP	Decreased GvHD, increased survival
Fransolet, Baron, 2016, Journal of hematology and oncology	Mice	M+F	BM transplantation + allogeneic splenocyte injection	Aza and DAC	3–16	DAC 0,75mg/kg, Aza 0,5 or 2 mg/kg	Day 10 2013Day 30, every 48 h	SC	Decreased GvHD, depending on dosing
Cooper, DiPersio, 2017, Journal of immunology	Mice	M	BM transplantation + allogeneic T cell injection	Aza	3–10	2 mg/kg	Day 15, 17, 19 and 21	IP	Decreased GvHD, this is Treg dependent
Ehx,Baron 2017 OncoImmunology	Mice	Unknown	Human PBMC injection (xeno–GvHD)	Aza	8–12	2 or 5 mg/kg	Day 1 – Day 21, every 48h	SC	Decreased GvHD, increased survival
Organ rejection									
Paluska Cihak 1982 Immunobiology*	Mice + Rats	M+F	Skin transplantation	Aza and DAC	4–11	6 or 20 mg/kg	Day -1, 1, 3, 5	IP	Prolonged graft survival, depending on dosing
Cheng C, Xia J, 2014, Immunology	Mice	M#	Cardiac transplantation	Aza	3–6	1 mg/kg	Daily untill end of exp.	IP	Aza alone did not prolong graft survival
Guo, Jiang, 2013, Transplant immunology	Mice	M	Cardiac transplantation	DAC	6	1,5 mg/kg	Day 1,2 and 3	IV	Prolonged graft survival
Wang X, Tao, 2017, Oncotarget^	Mice	M	Cardiac transplantation	DAC	Unknown	0,25 or 0,5 mg/kg	Daily for 14 days	IP	Prolonged graft survival
Uchida, Tisdale, 2014, PLOS ONE	Mice	M	Human stem cell transplantation (Xeno-rejection)	DAC	Unknown	0,5 μM (ex vivo)	48 h ex vivo prestimulation	Unknown	Prolonged graft survival
Hong J, Yang, 2013, Transplant immunology	Mice	Unknown	Islet transplantation	Aza	6-9	1 mg/kg	Day 0 and 1	Unknown	Aza alone did not prolong graft survival
Diabetes									
Zheng, Zhao, 2009, J mol med	Mice	M+F	NOD mice + cyclophosphamide injection	DAC	5	0,1 or 0,15 mg/kg	Daily for 5 days or 5 weeks	IP	Prevented diabetes
Wang, Shi, 2016, JCI insight	Mice	M	ob/ob mice + high fat diet	DAC	6–16	0,25 mg/kg	3x/week for 8 weeks	IP	Improved insulin sensitivity
Gao, Mu, 2019, Stem Cell Research and Therapy	Mice	M	C57BL/6 mice + high fat diet + STZ	DAC	18	0,25 mg/kg	Daily for 5 days	IP	DAC alone did not improve clinical outcome
Chen, Dong, 2019, Kidney international	Mice	M	db/db mice	DAC	6-9	1 mg/kg	2x/wk from week 8-20	IP	Improved diabetic nephropathy
Zhang, Liang, 2017, Kidney International	Mice	M	db/db mice	Aza	6	1 or 2 mg/kg	3x/week for 8 weeks	IP	Improved diabetic nephropathy, no effect on blood glucose levels
Atherosclerosis									
Cao, Xua , 2014, Endocrinology	Mice	M	ldlr mice + High fat and cholesterol diet	DAC	8	0,25 mg/kg	3x/week up to 30 weeks	IP	Decreased Atherosclerosis Development
Biliary Arthresia									
Li, Tang, 2016, the american physiological society	Mice	M+F	IP RVV injection	Aza	8–17	2,5 mg/kg	Day 1,2 and 3	IP	Decreased biliary arthresia and increased survival
Systemic Lupus Erythematodes (SLE)									
Yoshida, Izni, 1990, European journal of immunology	Mice	F	MRL-lpr mice	Aza	9–23	50 ug	2x/week from 6–21 weeks	IP	Increased survival and clinical outcome
Mizugaki, Nose, 1997, Clin Exp Immunol	Mice	Unknown	MRL-lpr mice	Aza	4–10	50 ug	2x/week from 4–20 weeks	IP	Increased survival and clinical outcome
Li, Tsokos, 2018, JCI Insight	Mice	F	MRL-lpr mice	Aza	4–6	5 μg (targeted to CD4+ cells)	every 10 days for 60 days	Unknown	Increased survival and clinical outcome in case of targeted delivery
Vitiligo									
Sreekumar, Smyth, 1996, Clin immunol immunopathol	Chicken	Unknown	Smyth line chicken	Aza	5–16	1 or 3 mg/kg	every 3 days up to 18 weeks	IP	Prevention of vitiligo in 1 strain, no effect in the other strain
Acute Respiratory Distress Syndrome (ARDS)									
Huang, Wang, 2016, Biomedicine & Pharmacotherapy	Mice	M	IP injection of LPS	Aza and DAC	8	1 mg/kg	1 h before LPS	IP	Alleviated lung injury
Cui, Shang, 2019, Laboratory Investigation	Mice	M	Inhalation of LPS	Aza	6–8	1 mg/kg	1 h after LPS	IP	Alleviated lung injury
Singer, D'alessio, 2014, Am Journal of Respiratory Cell and Molecular Biology	Mice	M	Inhalation of LPS	DAC	5–8	1 mg/kg	daily, starting 24 h after LPS	IP	Alleviated lung injury
Thangavel, Rajasingh, 2014, Am Journal of Pathology	Mice	M	IP injection of LPS	Aza	3–5	4,4 μmol/L/kg	1 h after LPS	IP	Aza alone did not increase survival
Thangavel, Rajasingh, 2015, Journal of Cell Science	Mice	M	IP injection of LPS	Aza	5	1 mg/kg	1 h after LPS	IP	Aza alone did not increase survival
Asthma									
Wu, Kuo, 2013, Allergy and Immunology	Mice	F	Injection and inhalation of ovalbumin	Aza	4–11	10,50,100 μg/dose	Every 3 days before OVA challenge	IP	Decreased airway hypersensitivity
Brand, Renz, 2012, Journal of Allergy and Clinical Immunology	Mice	F	Injection and inhalation of ovalbumin	DAC	4–6	0,2 mg/kg	Every 2 days before OVA challenge	IP	Decreased airway hypersensitivity
Rheumatoid Arthritis (RA)									
Kröger, Ehrlich, 1999, General Pharmacology	Rats	M	Injection of adjuvant in the paw	Aza	3	25 mg/kg	Day 3, 5, 7, 11, 15	IP	Aza partly seemed to decrease RA
Tóth, Rauch, 2019, Arthritis & Rheumatology	Mice	F	IP injection with proteoglycans	Aza	3–12	2 mg/kg	Every 2 days for 2 or 4 weeks	IP	Decreased RA
Multiple Sclerosis (MS)									
Chan, Wu, 2014, Molecular medicine	Mice	F	Injection of mog and heat-killed m. Tuberculosis	Aza	3–5	0,15 mg/kg/	Daily from Day-5 untill Day 10	IP	Protects against EAE
Wang X, Tao, 2017, Oncotarget ^	Mice	M	Injection of mog and heat-killed m. Tuberculosis	DAC	5	0,25 mg/kg	Daily from Day 3 or Day 10	IP	Protects against EAE, both prevention and treatment protocol
Mangano, Nicoletti, 2014, Journal of Cellular Physiology	Mice	M+F	Injection of mog or plp and heat-killed m. Tuberculosis	DAC	5–12	0,1 mg/kg	Daily from Day 0, Day 7 or from clinical onset	IP	Protects against EAE, both prevention and treatment protocol
Guillain Barré Syndrome (GBS)									
Fagone, Nicoletti, 2018, Journal of Neuroimmunology	Mice + Rats	M	Injection of p0 peptide and M. Tubercolosis	DAC	Unknown	0,1 mg/kg	Daily from Day 5 or from clinical onset for 21 days	IP	Decreased EAN, both prevention and treatment protocol

Two articles discuss different diseases and are therefore mentioned twice in the table. They are listed with * or ^

*M* male, *F* female, *IP* intraperitoneal, *IV* intravenous, *SC* subcutaneous, *GvHD* graft versus host disease, *LPS* lipopolysaccharides, *OVA* ovalbumin, *MOG* myelin oligodendrocyte glycoprotein, *PLP* proteolipid protein, *EAE* experimental autoimmune encephalomyelitis, *EAN* experimental autoimmune neuritis

### Study quality and risk of bias

**Fig. 2:**
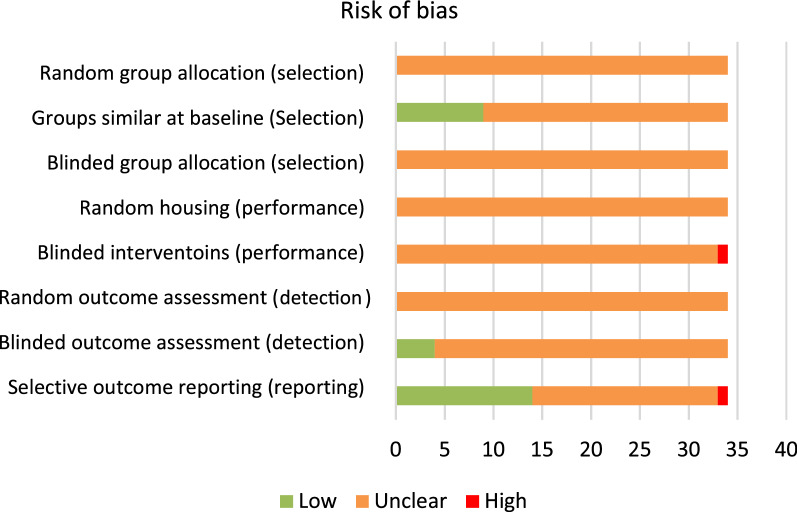
Reporting of Risk of Bias assessment of the included studies. The exact questions The following questions were asked: (1) Was the allocation sequence adequately generated and applied? (2) Were the groups similar at baseline or adjusted for confounders? (3) Was the allocation adequately concealed? (4) Were the animals randomly housed during the experiment? (5) Were the caregivers /investigators during the course of the experiment adequately blinded? (6) Were the animals selected at random during outcome assessment? (7) Was the outcome assessment adequately blinded? (8) Were incomplete data outcome adequately addressed? By reporting “yes” we indicated the study as low risk of bias, “no” as high risk of bias, and “unclear” as not reported

**Fig. 3:**
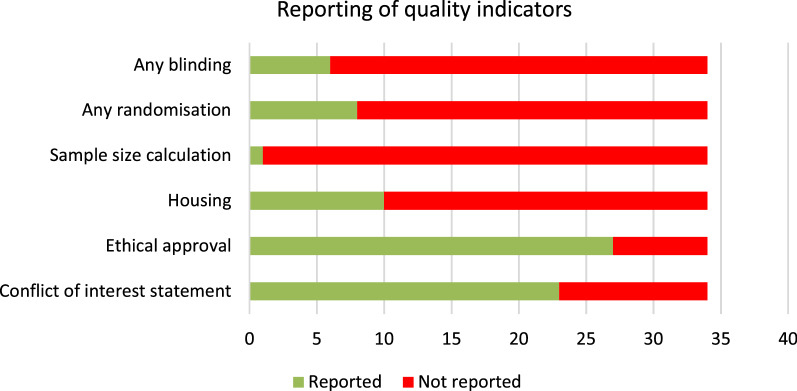
Reporting of quality indicators in the included studies. The following questions were asked: (1) Is any blinding reported? (2) Is any randomization reported? (3) Is the sample size calculation reported? (4) Are housing conditions reported? (5) Is ethical approval reported? (6) Are any conflicts of interest reported?

Results on risk of bias and on reporting of quality indicators are shown in Figs. 2 and 3, respectively. Scores per individual study can be found in Additional file 2: Figure S1. In 8 (23%) of the studies experiments were randomized in some way. However details regarding the procedure of randomization were not reported in any publication. This resulted in an unclear risk of bias for random allocation, random housing and randomization of outcome assessment. Any blinding of experiments was reported in 6 of the studies. Four of those reported blinded outcome assessment, which resulted in a low risk of bias. No blinding of group allocation or caregivers was reported, leading to an unclear risk of bias. One study scored a high risk of bias on blinding of the caregivers (performance bias), because it was stated that control animals did not receive any injections while experimental animals did. Baseline characteristics were properly reported and seemed comparable between groups in 9 studies, resulting in a low risk of bias for selection bias. The remaining studies did not report baseline characteristics and were therefore assigned an unclear risk of bias. Attrition bias was unclear in 20 of the studies: in these studies information about dropouts and animals used per group was reported insufficiently. In 14 cases information about drop-outs and number of animals used was properly mentioned, and no big differences in drop-outs between groups were seen, and the risk of attrition bias was therefore judged as low. In one publication a high risk of bias for attrition was scored because the number of animals used in total was not mentioned properly, while at the same time animals used varied a lot between experiments, which could indicate unreported dropouts. Sample size calculation was only mentioned once. Any statement about housing of animals was included in 10 studies. Ethical approval was reported 28 times. Any statement about conflict of interest was described in 24 of the studies.

### Data synthesis

#### Clinical outcomes

From the 35 included studies, 7 studied GvHD [19–25]. One of these studies also described organ rejection [19]. Five other studies also described organ rejection [26–30] of which one was addressed islet transplantation for the treatment of diabetes [30]. Since this study did not discuss the effect on the diabetes itself, we categorized it under ‘transplantation’. Organ or stem cell transplantation can be a lifesaving treatment for patients with organ failure or hematological malignancies. Major risks of transplantation are graft versus host disease (GvHD) and organ rejection [31, 32]. T-cells play a major role in both GvHD and organ rejection, and immune suppression is largely directed at dampening this type of response [33]. Most studies showed an improved outcome upon Aza or DAC treatment (Table 1), i.e. GvHD was decreased, and Aza/DAC prolonged graft survival in 4 out of 6 studies. In the other 2 studies, only a combination of Aza and another treatment led to prolonged survival.

Five studies described the effect of Aza/DAC on diabetes [34–38]. T1DM is characterized by the lack of insulin production in the pancreas. T1DM can be modeled in mice by inducing diabetes using streptozotocin (STZ) in C57BL/6 mice [30] or by accelerating the spontaneous occurrence of diabetes in non-obese diabetic (NOD) mice using cyclophosphamide (CY-NOD) [34]. The studies on diabetes had very divergent outcome measures, but had in common that 4 out of 5 studies improved clinical outcome. One study only showed improvement in combination with another drug.

Like diabetes, atherosclerosis is a consequence of obesity. Macrophage inflammation plays an important role in the atherosclerotic plaque formation, progression and rupture of the arteries [39]. One article described the effect of DAC on atherosclerosis [39] and showed reduced inflammation upon DAC treatment. One study described the effect of DAC of biliary arthresia, an autoimmune disease seen after viral infection of the liver soon after birth, and showed alleviation of the disease [40].

Systemic lupus erythematosus (SLE) is a chronic autoimmune disease characterized by the production of autoantibodies. Antibodies can be directed to all tissues and organs, but skin, joints, mucosa and kidneys are most often affected. The loss of self-tolerance is characterized by autoreactive T and B cells producing autoantibodies and causing tissue injury [41]. Several studies were found in which Aza was used to induce SLE [42, 43], but also 3 studies were found in which Aza was used as a potential treatment for SLE [44–46]. In these studies, Aza treatment resulted in increased survival and improved clinical outcome.

Like the induction of lupus in mice, chronic low-dose administration of Aza in chickens is known to induce auto-immune thyroiditis [47]. This was the only study in which a species other than mice or rats was used. The outcome was dependent on the strain. In one strain, vitiligo was prevented, while in the other strain no effect was seen.

Respiratory tract inflammatory conditions are widespread in the world. Respiratory tract inflammation is divided into acute and chronic respiratory inflammation. Acute inflammation includes pneumonia and acute respiratory distress syndrome (ARDS). The latter is an inflammatory disease initiated by a wide variety of both systemic (eg. sepsis) and pulmonary insults and leads to overwhelming inflammation in lung tissue [48]. Chronic respiratory tract inflammation includes (allergic) asthma and COPD. Asthma is a condition with chronic inflamed and narrowed airways and is triggered by allergies, cold, smoke, exercise and respiratory infections [49]. ARDS [50–54] and asthma [55, 56] were studied in 5 and 2 studies, respectively. In ARDS, 3 studies found alleviated lung injury. The 2 studies from Thangavel et al. only found an increase in survival when Aza was combined with other treatments. Both Aza and DAC decreased airway hypersensitivity in the 2 studies in asthma.

Rheumatoid arthritis (RA) is a systemic autoimmune disease with chronic inflammation of the membrane (synovium) that lines the joints which leads to destruction of the connective tissues like cartilage, tendons and bone. Treatment of RA consists of slowing down the degradation process and pain relief, but there is no cure [57]. There were two studies on RA and Aza [58, 59]. In 1 study Aza seemed to improve the clinical outcome, however no statistical analysis was performed. One study showed improved clinical outcome.

Multiple sclerosis (MS) and Guillain–Barré syndrome (GBS) are inflammatory autoimmune diseases causing demyelination of the nervous system. MS is a chronic disease affecting the central nervous system, while GBS can resolve over time and affects the peripheral nervous system [60, 61]. The effect of Aza in MS [28, 62, 63] and GBS [64] were examined in 3 and 1 studies, respectively. For MS all studies showed protection against experimental MS upon treatment with Aza/DAC. Two out of 3 studies showed that this was both the case employing a prevention as well as a treatment protocol. This was also the case for the study on GBS.

### Specific cell subsets and cytokines

Since Aza/DAC are used to treat inflammatory disorders, we wanted to know what the effect of these drugs was on different immune cell subsets and relevant cytokines. Aza/DAC is known to upregulate FOXP3, therefore special attention was given to the effect on Treg. Twenty-three studies described one or more cell subsets of which 17 studies reported on the effect of Aza/DAC on Treg (see Table 2).

**Table 2:** Reported cell subsets in the included studies including the effect of Aza/DAC on the reported cell subset

Author first, last, year, journal	Disease	FOXP3/Treg	CD45/Leukocytes	Granulocytes	Lymphocytes	Macrophages	CD3/Tcell	CD4/Thelper	CD8/Tcyt	B220/B-cells
Sánchez-Aberca, Pérez-Simon, 2010, The American Society of Hematology	GvHD	Equal or ↑								
Choi, Dipersio, 2010, The American Society of Hematology	GvHD	↑					↑			↑
Fransolet, Baron, 2016, Journal of Hematology and Oncology	GvHD	↑	↓		↓			Equal or ↓		
Cooper, DiPersio, 2017, Journal of Immunology	GvHD	↑					↑			↑
Ehx,Baron 2017 OncoImmunology	GvHD	↑								
Guo, Jiang, 2013, Transplant Immunology	Organ rejection	↑							↓	
Hong J, Yang, 2013, Transplant Immunology	Organ rejection	↑								
Zheng, Zhao, 2009, J Mol Med	Diabetes	↑						↑	↑	
Chen, Dong, 2019, Kidney International	Diabetes	↑						↓		
Cao, Xua, 2014, Endocrinology	Atherosclerosis							Equal	↓	↓
Li, Tang, 2016, The American Physiological Society	Biliary Arthresia	↑								
Yoshida, Izni, 1990, European Journal of Immunology	SLE							Equal	Equal	Equal
Li, Tsokos, 2018, JCI Insight	SLE	↑								
Singer, D'alessio, 2014, Am Journal of Respiratory Cell and Molecular Biology	ARDS	↑		↓						
Thangavel, Rajasingh, 2014, Am Journal of Pathology	ARDS			Equal						
Thangavel, Rajasingh, 2015, Journal of Cell Science	ARDS		Equal			Equal				
Wu, Kuo, 2012, Allergy and Immunology	Asthma	↑		↓	↓	↓				
Brand, Renz, 2011, Journal of Allergy and Clinical Immunology	Asthma		↓	↓	↓	↓				
Tóth, Rauch, 2019, Arthritis & Rheumatology	RA									↓
Chan, Wu, 2014, Molecular Medicine	MS	↑			↓					
Wang X, Tao, 2017, Oncotarget	MS	Equal or ↓	↓					↓	↓	
Mangano, Nicoletti, 2014, Journal of Cellular Physiology	MS	↑	↓					↓		
Fagone, Nicoletti, 2018, Journal of Neuroimmunology	GBS	↑	Equal				Equal	Equal	Equal	

In this table no differentiation is made between units (e.g. percentage or absolute cell number). Different techniques e.g. FACS, IHC) were used

All studies about GvHD and organ rejection showed an increase in Treg populations when mice were treated with Aza or DAC. In the study by Sanchez-Abarca et al., this was only the case with repeated dosing of Aza [21]. Also in the metabolic diseases diabetes and BA, Treg was increased upon Aza/DAC treatment. One study on SLE measured Treg, and showed an increase upon Aza treatment. In the lung diseases ARDS and asthma, the effect on Treg was measured in 2 studies; both found an increase in Treg. In all studies about neuroinflammatory diseases (MS, GBS) Treg were studied. Three studies found an increase in Treg, but 1 study showed equal percentages after DAC treatment and lowered absolute numbers of Treg.

Other cell subsets that are studied varied widely over the different studies. Studies that measured leukocytes/CD45 + cells (n = 5), granulocytes (n = 4), lymphocytes (n = 3) or macrophages (n = 3) showed a decrease or no change in these cell subsets. Looking further into the lymphocytes, the direction of the effect on CD3 + (n = − 3), CD4 + (n = 6), and CD8 + T cells (n = 4) and B220 B cells (n = 5) varied per study. The effect for the different cell types in general had the same direction, so when no effect was seen in CD4 + cells, also no effect was seen in CD8 + T cells and B220 cells. It has to be taken into account that for the summary of this data no differentiation was made between absolute cell numbers or percentages or between the methods of measurement (e.g. FACS/IHC etc.).

Seventeen studies looked at one or more cytokines. Most studies found reduced numbers or percentages of cytokines. Two studies showed no changes in any of the measured cytokines.

Table 3 shows a summary of data without differentiation between numbers/percentages and methods of measuring (e.g. FACS, ELISA/Luminex, qPCR). The pro-inflammatory cytokine IL-1β was measured in 7 studies. Five studies found lowered numbers/percentages of IL-1β, while 2 studies found no effect. The Th1-associated cytokines IL-12, IFN-γ and TNFα were lower in general. TNFα was measured in most studies, 11 in total. Eight studies showed lower TNFα levels, 2 showed no change and 1 study showed an increase. The Th2 associated cytokines IL-4, IL-5 and IL-13 were only measured in 3, 2 and 2 studies respectively. One study showed no effect on IL-4, the other studies showed a decrease in IL-4 and the other Th2 cytokines. The Th17 associated cytokines TGFβ, IL-6 and IL-17 were studied in 1, 7 and 6 studies respectively. All cytokines were equal of lower compared to control.

**Table 3:** Reported cytokines in the included studies including the effect of Aza/DAC on the reported cytokines

Author first, last, year, journal		mϕ/monocytes	Th1			Th2			Th17			Treg
	Disease	IL-1β	IL-12	IFN-γ	TNF-α	IL-4	IL-5	IL-13	TGF-β	IL-6	IL-17	IL-10
Ehx,Baron 2017 OncoImmunology	GvHD			↓	↓						Equal	
Wang, Shi, 2016, JCI Insight	Diabetes	↓			↓					↓		
Gao, Mu, 2019, Stem Cell Research and Therapy	Diabetes	Equal			Equal	Equal				Equal		Equal
Chen, Dong, 2019, Kidney International	Diabetes				↓							
Cao, Xua, 2014, Endocrinology	Atherosclerosis	↓			↓					↓		
Li, Tang, 2016, The American Physiological Society	Biliary arthresia										↓	
Li, Tsokos, 2018, JCI Insight	SE			*	*					*	*	*
Huang, Wang, 2016, Biomedicine & Pharmacotherapy	ARDS	↓			↓							
Cui, Shang, 2019, Laboratory Investigation	ARDS	↓			↓					↓		↑
Thangavel, Rajasingh, 2014, Am Journal of Pathology	ARDS				↑							
Thangavel, Rajasingh, 2015, Journal of Cell Science	ARDS	Equal			Equal					Equal		Equal
Wu, Kuo, 2012, Allergy and Immunology	Asthma					↓	↓	↓				↓
Brand, Renz, 2011, Journal of Allergy and Clinical Immunology	Asthma			↑		↓	↓	↓				
Chan, Wu, 2014, Molecular medicine	MS			↓							↓	
Wang X, Tao, 2017, Oncotarget	MS	↓	↓	↓	↓					↓	↓	
Mangano, Nicoletti, 2014, Journal of Cellular Physiology	MS			Equal					Equal		↓	Equal
Fagone, Nicoletti, 2018, Journal of Neuroimmunology	GBS			↓	↓					↓	↓	

In this table no differentiation is made between units (e.g. percentage, ng/ml or counts/mm). Different techniques (e.g. FACS, IHC, ELISA) were used

*Cytokines not compared to non-treated control. Targeted delivery lowers cytokines compared to empty nlg

The anti-inflammatory cytokine IL-10 was studied in 6 studies. 3 studies found no effect on IL-10 production. In these 3 studies also no effect on the pro-inflammatory cytokines was found, except for lowered IL-17 levels in one study. One study found an increase in IL-10 and 1 found a decrease in IL-10. One study used targeted delivery of Aza, using nanolipogels (nlg). In this study Aza-nlg lowered all cytokines including IL-10 compared to empty nlg.

## Discussion

Inflammatory disorders like diabetes [65], SLE [41], inflammatory lung diseases [48, 49], RA [57] and MS [60], but also rejection of transplanted organs [31] and GvHD [32] have a major effect on mortality, morbidity and costs of health care. These disorders have in common that they are often treated with immune suppressive drugs. Current classes of immune suppressive drugs never completely inhibit the inflammation and adverse side effects are common. Therefore there is a need for new drugs with other working mechanisms. Potential candidates are the DNMTi’s Aza and DAC [12, 21, 66]. Aza and DAC have been tested in several pre-clinical in vivo studies. In order to obtain an overview of disorders where Aza and/or DAC can be a potential treatment and to find out where information is lacking we systematically reviewed pre-clinical animal studies assessing Aza or DAC as a potential therapy for different inflammatory disorders.

Our review provides evidence that pre-clinically tested therapies seem to alleviate several inflammatory disorders. It is interesting to see that despite the large differences in diseases, disease models and treatment regimen, the overall conclusion is very similar. We show that Aza and DAC can improve health outcome in models for GvHD, organ rejection, diabetes, atherosclerosis, BA, SLE, vitiligo, ARDS, asthma, RA, MS and GBS.

Aza was developed in the sixties by the group of Vesely and Cihak as a cancerostatic drug [67]. In the eighties it was discovered by the same group that Aza has immune inhibitory effects when used in lower doses. Aza/DAC are used to treat hematological malignancies, with or without transplantation of stem cells. It therefore makes sense that the immune modulatory effect of Aza/DAC is most often studied in GvHD and the prevention of organ rejection. In the nineteen-eighties, Paluska et al. [19] and Sula et al. [68] (both from the group of Cihak), described for the first time that Aza and DAC have both immunosuppressive and immune stimulatory effects on allogeneic splenocyte induced GvHD. The effect was dependent on the dose and timing of Aza and DAC administration. More recent studies [21, 69–71] induced GvHD in mice by injecting bone-marrow derived stem cells (SCT) and allogeneic (Treg depleted) T cells in lethally irradiated mice. Aza/DAC was administered after SCT. Mice treated with Aza or DAC had increased survival rates and less signs of GvHD related symptoms. Cooper et al. showed that this effect of Aza was Treg dependent, using Treg depletion experiments [71].

The effect of Aza/DAC on organ rejection was studied with different organ transplantation models. Mice or rats were transplanted with skin [19], cardiac allograft [26–28], hematopoietic stem cell (HSC) [72] or pancreatic islets [30]. In the studies addressing the research question what the effect of Aza/DAC was on organ rejection, Aza/DAC was shown to prolong graft survival [19, 27–29]. However, in studies that examined whether Treg or Aza + rapamycin could prevent organ rejection, it appeared that treatment with Aza-treated Treg or monotreatment with Aza did not prolong graft survival. The latter two studies used Aza while the other studies (except Paluska et al., who used both) used DAC, however it cannot be concluded that the difference is due to the difference in Aza/DAC. Also, different doses and dosing regimen where used.

Five studies on diabetes were published [34–38], using 4 different mouse models. The STZ and CY-NOD models are used to study T1DM, while the db/db and ob/ob models are suited to study T2DM. The focus of the different studies was on insulin sensitivity, the effect on macrophage polarization and on diabetic nephropathy. The heterogeneity between the studies on seemingly the same subject is so large that it was not possible to make a good comparison. Still, except for the study of Gao et al., all studies showed improved clinical outcome. In the study of Gao, MSC in combination with DAC improved clinical outcome. DAC alone did not.

For ARDS the methodology used was more comparable. The disease is induced in mice by either injection or inhalation of LPS. Three studies show that a dose of 1 mg/kg of either Aza or DAC is effective in alleviating lung injury, either when injected 1 h before [50], 1 h after [51], or with repeated dosing of LPS [52]. In the 2 studies by Thangavel et al. a lethal dose of LPS was used. Aza alone did not increase survival, however in combination with Trichostatin A (TSA) a significant increase in survival was seen [73, 74]. In the second study a dose of 1 mg/kg was used like in the other ARDS studies.

For this systematic review we studied non-infectious inflammation including auto-immune and auto-inflammatory disorders, organ rejection after transplantation and GvHD. What these diseases have in common is that the immune system is over active and that the body is attacking its own tissues or the transplanted organ. Common treatment for these diseases is immunosuppression, often by suppressing T cells. It is very difficult, if not impossible, to make a comparison between the different diseases since the diseases are so divers.

The choice to measure which clinical outcome, but also which immune cells and which cytokines are mainly dependent on the specific disease and also on the hypothesis for the working mechanism of Aza/DAC. Inflammatory disorders can be roughly divided into Th1, Th2 and Th17 driven diseases. Unfortunately, not enough data on cells subsets and cytokine production are available to group results from the different diseases from the papers that we reviewed. Mainly, disease specific clinical outcome measures are shown for example. Studies about asthma will not show the effect on joint swelling, vice versa RA studies RA will not show results on lung inflammation.

When looking at the hypothesis, an interesting observation is that the hypothesis about the mechanism through which Aza/DAC could prevent or treat the different disorders varies widely among the studies. Fourteen studies hypothesized that induction of FOXP3, causing an increase in Treg, is the mechanism through which Aza/Dac can be used as a potential treatment for the different immune disorders. Seventeen studies measured FOXP3 and/or Treg, of which indeed most found an increase in FOXP3/Treg. Three studies hypothesized that a change in regulation of effector T cells/the Th1/Th2 balance [21, 56, 64] is affected by DNMTi, 3 mention a change in macrophage polarization [35, 36, 54], 1 study mentions a change in differentiation of CD34 + stem cells [29], 1 study regulation of neutrophil apoptosis by modulating DAPK1 expression [51] and 1 mentions a change in B-cells [59]. The remaining studies hypothesize that hypomethylation leads to an effect on specific target cells like podocytes [34] or lung epithelial cells [53] or just mention regulation by DNMTi’s in general. This also results in a wide variety of results that are described. Beside Treg, 18 studies in total mention changes in leukocytes, granulocytes, lymphocytes, macrophages, CD3−, CD4−, CD8− and B220 cells. This means that despite that the fact that all these studies are about immune disorders, 12 studies do not mention changes in the major subsets of immune cells. Like the immune cell subsets, the different cytokines are only mentioned in a few studies. One or more cytokines were measured in 17 studies. In general cytokine production is lower, except for IL-10, where contradictory results are found between studies and disorders. None of the studies describe mechanistically why cytokine levels change. The low number of studies describing cytokines prohibits conclusion on the effect of Aza/DAC on cytokine production.

Despite the differences in disease mechanism, hypothesis on the working mechanism and the differences in treatment, the effect on clinical outcome is very similar. Overall, in 31 out of the 35 included studies clinical outcome improved, while in 4 no effect was found. None on the studies showed that Aza/DAC deteriorated the disease. An important conclusion is that the effect of Aza and DAC seems to be dependent on the concentration that is used. A few studies tested different concentrations and saw that the effect was dose dependent. Beside different concentrations, also different dosing regimens were used. Most studies use multiple dosing, some start before the induction of the disorder (prevention protocol), while others start treating after the start/induction of the disorder (treatment protocol). Also a combination of treatment before and after induction is seen. In 4 of the ARDS studies a single dose of Aza/DAC is used. Because of the differences in dosing regimen it is not possible to draw a conclusion on which concentration is optimal, even though this is tested in several studies.

The different studies describe limited or no side effects in the animals treated with Aza/DAC. However, several studies also describe that Aza can induce the immune disease SLE in mice [42, 43] and vitiligo in chickens[47]. In these studies different strains were used, indicating that genetic background plays an important role in the outcome of the treatment. In human Aza/DAC are well-tolerated and side effects like induction of SLE or vitiligo are not seen.

### Limitations

Inflammatory disorders form a broad spectrum of disorders and conclusions on the effect of Aza/DAC in a given disorder should not be drawn based on the outcome in another. For every disease only a limited number of studies was found. The heterogeneity in experimental approach both within and between diseases was substantial. Dosing and dosing schedule were not comparable between different studies. Taken together, this did not allow for a meta-analysis. Therefore, the interpretation of the results had to be based on the results as reported by the authors and the effect size could not be taken into account. Also, some studies used a prevention protocol while others used a treatment protocol. Except for organ transplantation/GvHD a preventive protocol is often not possible. This limits the possibility to translate the study to the human situation.

Like in many other systematic reviews of animal studies, an important limitation of this review is that many of the essential methodological details of animal studies included in our review were poorly reported [75]. This directly affects the reliability of the conclusions based on those studies and hampers a reliable risk of bias assessment. Despite the fact that we cannot reliably estimate how internally valid the results are, we included all papers that met the criteria, since poor reporting does not necessarily mean impaired methodology. Poor reporting emphasizes the fact that reporting should be improved for future studies by reporting more essential details, as described for example in the ARRIVE guidelines [76], as it can only increase the reliability of a study.

The limited data available also did not allow for an evaluation of publication bias. It is, however, remarkable that studies studying a combination of Aza/DAC with another therapy found no effect for Aza/DAC single treatment, while the studies that primarily studied the effect of Aza/DAC on the disease always found an improved clinical outcome.

Another important thing to take into account when interpreting the results is that very divergent data are displayed. For example, the absolute number of cells may decrease, but the percentage of cells may increase. Also results from for example qPCR and ELISA/Luminex can differ in effect size. In Tables 2 and 3 we combined all data without looking at the method of measurement or the derived quantity. It is difficult to say what the most important or most reliable outcome measure is. Describing both changes in absolute numbers and changes in percentages would be optimal. What method is best, depends on the exact research question.

### Clinical relevance and recommendations

Aza and DAC are currently used for the treatment of myelodysplastic diseases such as AML and MDS [5, 6]. Clinical studies are already done to test whether Aza/DAC may also improve stem cell transplantation in these diseases [77–80]. To our knowledge, no clinical studies are done to assess whether Aza/DAC can be used to prevent GvHD or rejection of transplanted organs or as a treatment for diabetes, atherosclerosis, BA, SLE, vitiligo, ARDS, asthma, RA, MS or GBS. Based on the results of our review, GvHD/organ transplantation and inflammatory lung diseases seem interesting candidates for clinical studies. However, conclusions from this review are based on limited data and should therefore be used with caution.

Aza is already used in hematological malignancies. Therefore it can be considered safe in humans. A big challenge will be to apply the best dose. More experiments in animals can help to optimize the design for a clinical study. For clinical studies it is important to have biomarkers that can be measured to assess the result of the therapy, preferably a marker that can be measured in blood. We therefore recommend that for future animal studies using Aza/DAC to treat inflammatory disease, more immune related outcomes, such as changes in cell subsets and cytokines are being measured. Following this, it would be very useful to investigate whether for these biomarkers the results of the pre-clinical studies are in line with future clinical studies.

## Conclusions

Aza and DAC seem to be promising candidates for the therapy of different inflammatory disorders. Increased Treg numbers seems to be the common ground for this effect. Based on these results it is too early to start clinical studies. Improving the reporting and methodological quality of animal studies can help to advance the transition to clinical studies. Further pre-clinical studies should include more biomarkers, such as immune cells subsets and cytokines, this will facilitate the step to clinical studies.

## Supplementary Information

**Additional file 1: Table S1.** Search strategy.

**Additional file 2: Figure S1.** Risk of Bias assessment and reporting of quality indicators of the included studies per study.

## Data Availability

All included data are available in the public domain and all references are included in our reference list. Extracted data will be made available to individual scientists upon reasonable request.

## Abbreviations

Aza: Azacytidine
DAC: 5-Aza 2′deoxycitidine
GvHD: Graft versus host disease
Treg: Regulatory T cell
DNMT: DNA methyltransferases
MDS: Myelodysplastic syndrome
AML: Acute myeloid leukemia
Tconv: Conventional T cells
TSDR: Treg-specific demethylated region
SLE: Systemic lupus erythematosus
RA: Rheumatoid arthritis
MS: Multiple sclerosis
GBS: Guillain–Barré syndrome
Nlg: Nanolipogels
