# Supplementary material for: Immune responses to azacytidine in animal models of inflammatory disorders: a systematic review
Source: J Transl Med. 2021 Jan 6;19:11. doi: 10.1186/s12967-020-02615-2 (PMC7788785; doi:10.1186/s12967-020-02615-2)
Supplement: Supplementary file 1 — Additional file 1: Table S1. Search strategy. [file 12967_2020_2615_MOESM1_ESM.pdf]

Supplementary table 1: search strategy

Pubmed	Search strategy
Azacytidine or Decitabine	"Azacitidine"[Mesh] OR Decitabine [supplementary concept] OR Azacytidine [Tiab] OR Azacitidine [Tiab] OR 5-Azacytidine [Tiab] OR Vidaza [Tiab] OR NSC 102816 [Tiab] OR NSC102816 [Tiab] OR Decitabine [Tiab] OR AzadC compound [Tiab] OR 5-azadeoxycytidine [Tiab] OR 5-deoxyazacytidine [Tiab] OR 5-Aza [Tiab] OR 2'-deoxy-5-azacytidine [Tiab] OR NSC 127716 [Tiab] OR Dacogen [Tiab] OR 5azacyd [Tiab] OR Azacitidin [Tiab] OR Ladakamycin [Tiab] OR Myolosar [Tiab] OR u 18496 [Tiab] OR u18496 [Tiab] OR nsc127716 [Tiab] OR 5 azadesoxycytidine [Tiab]
Inflammatory disorders	"Myositis"[Mesh] OR "immune system diseases"[Mesh] OR "digestive system diseases"[Mesh] OR "skin and connective tissue diseases "[Mesh] OR "Diabetes Mellitus"[Mesh] OR "Spondylarthropathies"[Mesh] OR "Sialadenitis"[Mesh] OR "Sarcoidosis"[Mesh] OR "Myocarditis"[Mesh] OR "Postpericardiotomy Syndrome"[Mesh] OR "Endocarditis"[Mesh] OR "Cystitis"[Mesh] OR "Antisynthetase syndrome" [Supplementary Concept] OR "Autoimmune oophoritis" [Supplementary Concept] OR "Anemia"[Mesh] OR "Endometriosis"[Mesh] OR "Orchitis"[Mesh] OR "Evans Syndrome" [Supplementary Concept] OR "Anemia, Pernicious"[Mesh] OR "Red Cell Aplasia, Pure"[Mesh] OR "Thrombocytopenia"[Mesh] OR "Fasciitis"[Mesh] OR "Mikulicz' Disease"[Mesh] OR "Orbital Pseudotumor"[Mesh] OR "Sinusitis"[Mesh] OR "Tuberculosis"[Mesh] OR "Plasma Cell Granuloma, Pulmonary"[Mesh] OR "Retroperitoneal Fibrosis"[Mesh] OR "Mediastinal Fibrosis" [Supplementary Concept] OR "Pyelonephritis, Xanthogranulomatous"[Mesh] OR "Nephritis, Interstitial"[Mesh] OR "Facial Hemiatrophy"[Mesh] OR "Brachial Plexus Neuritis"[Mesh] OR "Isaacs Syndrome"[Mesh] OR "Paraneoplastic Cerebellar Degeneration"[Mesh] OR "Hashimoto's encephalitis" [Supplementary Concept] OR "Restless Legs Syndrome"[Mesh] OR "Chorea"[Mesh] OR "Paraneoplastic Syndromes, Ocular"[Mesh] OR "Uveitis"[Mesh] OR "Opsoclonus-Myoclonus Syndrome"[Mesh] OR "Optic Neuritis"[Mesh] OR "Scleritis"[Mesh] OR "Susac Syndrome"[Mesh] OR "Tolosa-Hunt Syndrome"[Mesh] OR "Meniere Disease"[Mesh] OR "Vasculitis"[Mesh] OR "Host vs Graft reaction"[Mesh] OR "Graft vs Host Reaction"[Mesh] OR "Osteitis"[Mesh] OR "Bursitis"[Mesh] OR "Synovitis"[Mesh] OR "Radiculopathy"[Mesh] OR "Allergy and immunology"[Mesh] OR immune [Tiab] OR Goodpasture syndrome[Tiab] OR Addison [Tiab] OR Anemia [Tiab] OR Anaemia [tiab] OR Polyangiitis [Tiab] OR Churg-Strauss Syndrome [Tiab] OR Antiphospholipid Syndrome[Tiab] OR Arthritis [Tiab] OR Arthropathies [Tiab] OR Arthropathy [Tiab] OR Polyarthritis [tiab] OR Polyarthritides [tiab] OR Caplan [tiab] OR Caplan's [tiab] OR Felty [tiab] OR Felty's [tiab] OR Sjogrens [tiab] OR Sjogren's [tiab] OR Sicca Syndrome [tiab] OR Still's [tiab] OR Stills Disease [tiab] OR Hypophysitis[Tiab] OR Lymphoproliferative Syndrome [Tiab] OR Canale-Smith [tiab] OR Dermatitis [Tiab] OR Diabetes [Tiab] OR IDDM [Tiab] OR Graves [Tiab] OR Hepatitis [Tiab] OR Dermatosis [Tiab] OR Lupus [Tiab] OR Ophthalmia [Tiab] OR Pemphigus [Tiab] OR Pemphigoid [Tiab] OR Polyendocrinopathies [Tiab] OR Purpura [Tiab] OR Encephalitis[Tiab] OR Sclerosis [Tiab] OR Encephalomyelitis [Tiab] OR Leukoencephalitis [Tiab] OR Neuromyelitis Optica[Tiab] OR Myelitis [Tiab] OR Lambert-Eaton [Tiab] OR Myasthenia Gravis[Tiab] OR Encephalomyelitis [Tiab] OR Neuritis [Tiab] OR Polyradiculoneuropathy[Tiab] OR Guillain-Barre [Tiab] OR Miller Fisher [Tiab] OR Stiff-Person [Tiab] OR Uveomeningoencephalitic Syndrome[Tiab] OR Vogt–Koyanagi–Harada disease [Tiab] OR Vasculitis [Tiab] OR Arteritis [Tiab] OR Myositis [Tiab] OR Lichenoid Eruptions[Tiab] OR Lichen [Tiab] OR Pityriasis Lichenoides[Tiab] OR Psoriasis[Tiab] OR Psoriases [Tiab] OR

	Pustulosis [Tiab] OR Psoriatic [Tiab] OR Rheumatoid [Tiab] OR Wissler's [Tiab] OR Rheumatic [Tiab] OR Scleroderma [Tiab] OR CREST Syndrome[Tiab] OR Colitis [Tiab] OR Crohn [Tiab] OR Crohn's [Tiab] OR Crohns [Tiab] OR Spondylarthropathies[Tiab] OR Spondylarthropathy [Tiab] OR Ankylosing Spondylitis [Tiab] OR Sialadenitis[Tiab] OR Parotitis[Tiab] OR Thyroiditis [Tiab] OR Hashimoto Disease[Tiab] OR Panniculitis[Tiab] OR Sarcoidosis[Tiab] OR Uveoparotid Fever[Tiab] OR Enteritis [Tiab] OR Ileocolitis [Tiab] OR Ileitis [Tiab] OR Myocarditis[Tiab] OR Postpericardiotomy Syndrome [Tiab] OR Endocarditis [Tiab] OR Cystitis [Tiab] OR Liver Cirrhosis [Tiab] OR Sclerosing Cholangitis [Tiab] OR antisynthetase syndrome[Tiab] OR Alopecia Areata[Tiab] OR Angioedema [Tiab] OR dermatitis[Tiab] OR Hives[Tiab] OR Asthma[Tiab] OR Asthmas [Tiab] OR Epidermolysis Bullosa Acquisita[Tiab] OR Erythema Nodosum[Tiab] OR Hidradenitis Suppurativa[Tiab] OR Vitiligo [Tiab] OR Polyendocrinopathies [Tiab] OR polyendocrinopathy [Tiab] OR oophoritis [Tiab] OR Endometriosis [Tiab] OR Orchitis[Tiab] OR enteropathy[Tiab] OR enteropathies [tiab] OR Celiac Disease [Tiab] OR sprue [Tiab] OR Coeliac disease [Tiab] OR Evans Syndrome[Tiab] OR Cryoglobulinemia[Tiab] OR Red-Cell Aplasia [Tiab] OR Thrombocytopenia [Tiab] OR Adiposis Dolorosa[Tiab] OR Fasciitis[Tiab] OR Mikulicz' Disease[Tiab] OR Orbital Pseudotumor[Tiab] OR Sinusitis[Tiab] OR Tuberculosis[Tiab] OR Plasma Cell Granuloma, Pulmonary[Tiab] OR Fibrosis [Tiab] OR Nephritis [Tiab] OR Angiolymphoid Hyperplasia with Eosinophilia[Tiab] OR Mixed Connective Tissue Disease[Tiab] OR rheumatism[Tiab] OR Facial Hemiatrophy[Tiab] OR Neuritis[Tiab] OR Polychondritis [Tiab] OR Schnitzler Syndrome[Tiab] OR Fibromyalgia[Tiab] OR Isaacs Syndrome[Tiab] OR Paraneoplastic Cerebellar Degeneration[Tiab] OR Hashimoto's encephalitis[Tiab] OR Restless Legs Syndrome[Tiab] OR Sydenham [Tiab] OR Uveitis[Tiab] OR Cogan Syndrome[Tiab] OR Plasminogen Deficiency, Type I[Tiab] OR Opsoclonus-Myoclonus Syndrome[Tiab] OR Scleritis[Tiab] OR Susac Syndrome[Tiab] OR Tolosa-Hunt Syndrome[Tiab] OR Behcet Syndrome[Tiab] OR Meniere Disease[Tiab] OR Mucocutaneous Lymph Node Syndrome[Tiab] OR Polymyalgia Rheumatica[Tiab] OR Vasculitis[Tiab] OR Postmyocardial infarction syndrome[Tiab] OR Ord's thyroiditis[Tiab] OR neutropenia[Tiab] OR Eosinophilic fascilitis[Tiab] OR IgG4 related disease[Tiab] OR Acute motor axonal neuropathy[Tiab] OR Mooren's ulcer[Tiab] OR inner ear disease[Tiab] OR pancreatitis[Tiab] OR immunoglobulin G4 related disease [Tiab] OR IPEX syndrome [Tiab] OR antiphospholipid syndrome [tiab] OR immunoglobulin A nephropathy [Tiab] OR IgA nephropathy [Tiab] OR psorasiasiform [Tiab] OR willan lepra [Tiab] OR regional enterocolitis [Tiab] OR cleron disease [Tiab] OR Beauvais Disease [Tiab] OR arhrochondritis [Tiab] OR chariot disease [Tiab] OR enthesitis [Tiab] OR osteitis [tiab] OR bursitis [Tiab] OR synovitis [Tiab] OR Blau syndrome [tiab] OR allergy [tiab] OR eczema [tiab] OR radiculopathy [Tiab] OR Radiculitis [Tiab] or Gastritis [Tiab] OR esophagitis [Tiab] OR Graft vs Host [Tiab] OR Graft versus host [Tiab] OR Host vs Graft [Tiab] OR Host versus Graft [Tiab] OR Rejection[Tiab] OR Rejections [Tiab] OR Inflammatory disease*[Tiab] OR Runt disease [Tiab] OR Homologous Wasting [tiab] OR Inflammation [tiab] OR inflammatory [tiab] OR runting disease [Tiab] OR runting syndrome [Tiab] OR GVH disease [Tiab] OR allogeneic disease [Tiab] OR transplantation reaction [Tiab]
Animal studies	(("animal experimentation"[MeSH Terms] OR "models, animal"[MeSH Terms] OR "invertebrates"[MeSH Terms] OR "Animals"[Mesh:noexp] OR "animal population groups"[MeSH Terms] OR "chordata"[MeSH Terms:noexp] OR "chordata, nonvertebrate"[MeSH Terms] OR "vertebrates"[MeSH Terms:noexp] OR "amphibians"[MeSH Terms] OR "birds"[MeSH Terms] OR "fishes"[MeSH Terms] OR "reptiles"[MeSH Terms] OR "mammals"[MeSH Terms:noexp] OR "primates"[MeSH

	Terms:noexp] OR "artiodactyla"[MeSH Terms] OR "carnivora"[MeSH Terms] OR "cetacea"[MeSH Terms] OR "chiroptera"[MeSH Terms] OR "elephants"[MeSH Terms] OR "hyraxes"[MeSH Terms] OR "insectivora"[MeSH Terms] OR "lagomorpha"[MeSH Terms] OR "marsupialia"[MeSH Terms] OR "monotremata"[MeSH Terms] OR "perissodactyla"[MeSH Terms] OR "rodentia"[MeSH Terms] OR "scandentia"[MeSH Terms] OR "sirenia"[MeSH Terms] OR "xenarthra"[MeSH Terms] OR "haplorhini"[MeSH Terms:noexp] OR "strepsirhini"[MeSH Terms] OR "platyrrhini"[MeSH Terms] OR "tarsii"[MeSH Terms] OR "catarrhini"[MeSH Terms:noexp] OR "cercopithecidae"[MeSH Terms] OR "hylobatidae"[MeSH Terms] OR "hominidae"[MeSH Terms:noexp] OR "gorilla gorilla"[MeSH Terms] OR "pan paniscus"[MeSH Terms] OR "pan troglodytes"[MeSH Terms] OR "pongo pygmaeus"[MeSH Terms]) OR ((animals[tiab] OR animal[tiab] OR mice[Tiab] OR mus[Tiab] OR mouse[Tiab] OR murine[Tiab] OR woodmouse[tiab] OR rats[Tiab] OR rat[Tiab] OR murinae[Tiab] OR muridae[Tiab] OR cottonrat[tiab] OR cottonrats[tiab] OR hamster[tiab] OR hamsters[tiab] OR cricetinae[tiab] OR rodentia[Tiab] OR rodent[Tiab] OR rodents[Tiab] OR pigs[Tiab] OR pig[Tiab] OR swine[tiab] OR swines[tiab] OR piglets[tiab] OR piglet[tiab] OR boar[tiab] OR boars[tiab] OR "sus scrofa"[tiab] OR ferrets[tiab] OR ferret[tiab] OR polecat[tiab] OR polecats[tiab] OR "mustela putorius"[tiab] OR "guinea pigs"[Tiab] OR "guinea pig"[Tiab] OR cavia[Tiab] OR callithrix[Tiab] OR marmoset[Tiab] OR marmosets[Tiab] OR cebuella[Tiab] OR hapale[Tiab] OR octodon[Tiab] OR chinchilla[Tiab] OR chinchillas[Tiab] OR gerbillinae[Tiab] OR gerbil[Tiab] OR gerbils[Tiab] OR jird[Tiab] OR jirds[Tiab] OR merione[Tiab] OR meriones[Tiab] OR rabbits[Tiab] OR rabbit[Tiab] OR hares[Tiab] OR hare[Tiab] OR diptera[Tiab] OR flies[Tiab] OR fly[Tiab] OR dipteral[Tiab] OR drosophila[Tiab] OR drosophilidae[Tiab] OR cats[Tiab] OR cat[Tiab] OR carus[Tiab] OR felis[Tiab] OR nematoda[Tiab] OR nematode[Tiab] OR nematodes[Tiab] OR sipunculida[Tiab] OR dogs[Tiab] OR dog[Tiab] OR canine[Tiab] OR canines[Tiab] OR canis[Tiab] OR sheep[Tiab] OR sheeps[Tiab] OR mouflon[Tiab] OR mouflons[Tiab] OR ovis[Tiab] OR goats[Tiab] OR goat[Tiab] OR capra[Tiab] OR capras[Tiab] OR rupicapra[Tiab] OR chamois[Tiab] OR haplorhini[Tiab] OR monkey[Tiab] OR monkeys[Tiab] OR anthropoidea[Tiab] OR anthropoids[Tiab] OR saguinus[Tiab] OR tamarin[Tiab] OR tamarins[Tiab] OR leontopithecus[Tiab] OR hominidae[Tiab] OR ape[Tiab] OR apes[Tiab] OR "pan paniscus"[Tiab] OR bonobo[Tiab] OR bonobos[Tiab] OR "pan troglodytes"[Tiab] OR gibbon[Tiab] OR gibbons[Tiab] OR siamang[Tiab] OR siamangs[Tiab] OR nomascus[Tiab] OR symphalangus[Tiab] OR chimpanzee[Tiab] OR chimpanzees[Tiab] OR prosimian[Tiab] OR prosimians[Tiab] OR "bush baby"[Tiab] OR bush babies[Tiab] OR galagos[Tiab] OR galago[Tiab] OR pongidae[Tiab] OR gorilla[Tiab] OR gorillas[Tiab] OR "pongo pygmaeus"[Tiab] OR orangutan[Tiab] OR orangutans[Tiab] OR lemur[Tiab] OR lemurs[Tiab] OR lemuridae[Tiab] OR horse[Tiab] OR horses[Tiab] OR equus[Tiab] OR cow[Tiab] OR calf[Tiab] OR bull[Tiab] OR chicken[Tiab] OR chickens[Tiab] OR gallus[Tiab] OR quail[Tiab] OR bird[Tiab] OR birds[Tiab] OR quails[Tiab] OR poultry[Tiab] OR poultries[Tiab] OR fowl[Tiab] OR fowls[Tiab] OR reptile[Tiab] OR reptilia[Tiab] OR reptiles[Tiab] OR snakes[Tiab] OR snake[Tiab] OR lizard[Tiab] OR lizards[Tiab] OR alligator[Tiab] OR alligators[Tiab] OR crocodile[Tiab] OR crocodiles[Tiab] OR turtle[Tiab] OR turtles[Tiab] OR amphibian[Tiab] OR amphibians[Tiab] OR amphibia[Tiab] OR frog[Tiab] OR frogs[Tiab] OR bombina[Tiab] OR salientia[Tiab] OR toad[Tiab] OR toads[Tiab] OR "epidalea calamita"[Tiab] OR salamander[Tiab] OR salamanders[Tiab] OR eel[Tiab] OR eels[Tiab] OR fish[Tiab] OR fishes[Tiab] OR pisces[Tiab] OR catfish[Tiab] OR catfishes[Tiab] OR siluriformes[Tiab] OR arius[Tiab] OR heteropneustes[Tiab] OR sheatfish[Tiab] OR perch[Tiab] OR

	perches[Tiab] OR percidae[Tiab] OR perca[Tiab] OR trout[Tiab] OR trouts[Tiab] OR char[Tiab] OR chars[Tiab] OR salvelinus[Tiab] OR minnow[Tiab] OR cyprinidae[Tiab] OR carps[Tiab] OR carp[Tiab] OR zebrafish[Tiab] OR zebrafishes[Tiab] OR goldfish[Tiab] OR goldfishes[Tiab] OR guppy[Tiab] OR guppies[Tiab] OR chub[Tiab] OR chubs[Tiab] OR tinca[Tiab] OR barbels[Tiab] OR barbus[Tiab] OR pimephales[Tiab] OR promelas[Tiab] OR "poecilia reticulata"[Tiab] OR mullet[Tiab] OR mullets[Tiab] OR eel[Tiab] OR eels[Tiab] OR seahorse[Tiab] OR seahorses[Tiab] OR mugil curema[Tiab] OR atlantic cod[Tiab] OR shark[Tiab] OR sharks[Tiab] OR catshark[Tiab] OR anguilla[Tiab] OR salmonid[Tiab] OR salmonids[Tiab] OR whitefish[Tiab] OR whitefishes[Tiab] OR salmon[Tiab] OR salmons[Tiab] OR sole[Tiab] OR solea[Tiab] OR lamprey[Tiab] OR lampreys[Tiab] OR pumpkinseed[Tiab] OR sunfish[Tiab] OR sunfishes[Tiab] OR tilapia[Tiab] OR tilapias[Tiab] OR turbot[Tiab] OR turbots[Tiab] OR flatfish[Tiab] OR flatfishes[Tiab] OR sciuridae[Tiab] OR squirrel[Tiab] OR squirrels[Tiab] OR chipmunk[Tiab] OR chipmunks[Tiab] OR suslik[Tiab] OR susliks[Tiab] OR vole[Tiab] OR voles[Tiab] OR lemming[Tiab] OR lemmings[Tiab] OR muskrat[Tiab] OR muskrats[Tiab] OR lemmus[Tiab] OR otter[Tiab] OR otters[Tiab] OR marten[Tiab] OR martens[Tiab] OR martes[Tiab] OR weasel[Tiab] OR badger[Tiab] OR badgers[Tiab] OR ermine[Tiab] OR mink[Tiab] OR minks[Tiab] OR sable[Tiab] OR sables[Tiab] OR gulo[Tiab] OR gulos[Tiab] OR wolverine[Tiab] OR wolverines[Tiab] OR mustela[Tiab] OR llama[Tiab] OR llamas[Tiab] OR alpaca[Tiab] OR alpacas[Tiab] OR camelid[Tiab] OR camelids[Tiab] OR guanaco[Tiab] OR guanacos[Tiab] OR chiroptera[Tiab] OR chiropteras[Tiab] OR bat[Tiab] OR bats[Tiab] OR fox[Tiab] OR foxes[Tiab] OR iguana[Tiab] OR iguanas[Tiab] OR xenopus laevis[Tiab] OR parakeet[Tiab] OR parakeets[Tiab] OR parrot[Tiab] OR parrots[Tiab] OR donkey[Tiab] OR donkeys[Tiab] OR mule[Tiab] OR mules[Tiab] OR zebra[Tiab] OR zebras[Tiab] OR shrew[Tiab] OR shrews[Tiab] OR bison[Tiab] OR bisons[Tiab] OR buffalo[Tiab] OR buffaloes[Tiab] OR deer[Tiab] OR deers[Tiab] OR bear[Tiab] OR bears[Tiab] OR panda[Tiab] OR pandas[Tiab] OR "wild hog"[Tiab] OR "wild boar"[Tiab] OR fitchew[Tiab] OR fitch[Tiab] OR beaver[Tiab] OR beavers[Tiab] OR jerboa[Tiab] OR jerboas[Tiab] OR capybara[Tiab] OR capybaras[Tiab] OR canine[tiab] OR bovine[tiab] OR porcine[tiab] OR hog[tiab] OR hogs[tiab]) NOT medline[sb]))

**Embase**	**Search strategy**
Azacytidine or Decitabine	exp decitabine/ OR exp azacitidine/ OR (Azacytidine OR Azacitidine OR 5-Azacytidine OR Vidaza OR NSC 102816 OR NSC102816 OR Decitabine OR AzadC compound OR 5-azadeoxycytidine OR 5-deoxyazacytidine OR 5-aza-2'-deoxycytidine OR 5-Aza OR 2'-deoxy-5-azacytidine OR NSC 127716 OR Dacogen OR mylosar OR 5 azacitidine OR 5 azacyd OR 5 azacytidin OR 5 azacytidine OR azacitidin OR ladakamycin OR u 18496 OR nsc 127716 OR nsc127716 OR u18496).ti,ab.
Inflammatory disorders	Exp immunopathology/ or Exp abdominal cavity inflammation/ or exp cardiovascular inflammation / or exp dermatitis/ or exp digestive system inflammation/ or exp experimental inflammation/ or exp foreign body reaction/ or exp granulomatous inflammation/ or exp inflammation of the eye and surrounding structures/ or mouth inflammation/ or exp musculoskeletal system inflammation/ or exp inflammatory disease/ or exp sialoadenitis/ or exp optic neuritis/ or nervous system inflammation/ or exp respiratory tract inflammation/ or exp urogenital tract inflammation/ or exp cystitis/ or exp nephritis/ or exp diabetes Mellitus/ or exp lichenoid eruption/ or psoriasis/ or exp rheumatic fever/ or exp

	spondyarthropathies/ or exp postpericardiotomy syndrome/ or exp liver cirrhosis/ or exp alopecia Areata/ or exp angioneurotic edema/ or exp Urticaria/ or exp lupus erythematosus/ or exp epidermolysis bullosa acquisita/ or exp hidradenitis suppurativa/ or exp vittiligo/ or exp polyendocrinpathy/ or exp ovary inflammation/ or exp celiac disease/ or exp anemia/ or exp endometriosis/ or exp orchitis/ or exp autoimmune hemolytic anemia/ or exp cryoglobulinaemia/ or exp thrombocytopenia/ or exp orbital pseudotumor/ or adiposis Dolorosa/ or exp Mikulicz disease/ or exp tuberculosis/ or exp lung granuloma/ or exp retroperitoneal fibrosis/ or exp angiofollicular lymph node hyperplasia/ or exp mixed connective tissue disease/ or exp hemifacial atrophy/ or exp branchial plexus neuropathy/ or exp fibromyalgia/ or restless legs syndrome/ or exp chorea/ or exp paraneoplastic syndrome/ or exp scleritis/ or exp susac syndrome/ or exp tolosa hunt syndrome/ or exp meniere disease/ or exp mucocutaneous lymph node syndrome/ or exp rheumatic polymyalgia/ or exp allergy/ or (Sialadenitis OR Schnitzler Syndrome OR abdominal cavity inflammation OR cardiovascular inflammation OR digestive system inflammation OR experimental inflammation OR foreign body reaction OR granulomatous inflammation OR inflammation of the eye and surrounding structures OR musculoskeletal system inflammation OR uveitis OR sialoadenitis OR chondritis OR nervous system inflammation OR respiratory tract inflammation OR diabetes OR lichenoid eruption OR angioneurotic edema OR Angioedema OR Urticaria OR polyendocrinopathy OR polyendocrinopathies OR Cellulitis OR ovary inflammation OR orchitis OR cryoglobulinaemia OR Mikulicz disease OR lung granuloma OR hemifacial atrophy OR branchial plexus neuropathy OR Facial Hemiatrophy OR Brachial Plexus Neuritis OR paraneoplastic syndrome OR Autoimmune diseases OR autoimmune disease OR Auto-immune disease OR Auto-immune diseases OR immune OR auto-immune OR autoimmune OR Goodpasture syndrome OR Addison OR Anemia OR Polyangiitis OR Churg-Strauss Syndrome OR Antiphospholipid Syndrome OR Arthritis OR Arthropathies OR Polyarthritis OR Polyarthritides OR Caplan OR Caplan's OR Felty OR Felty's OR Sjogrens OR Sjogren's OR Sicca Syndrome OR Still's OR Stills Disease OR Hypophysitis OR Lymphoproliferative Syndrome OR Canale-Smith OR Dermatitis OR diabetes OR IDDM OR Graves OR Hepatitis OR Dermatosis OR Lupus OR Ophthalmia OR Pemphigus OR Pemphigoid OR Polyendocrinopathies OR Purpura OR Encephalitis OR Sclerosis OR Encephalomyelitis OR Leukoencephalitis OR Neuromyelitis Optica OR Myelitis OR Lambert-Eaton OR Myasthenia Gravis OR Encephalomyelitis OR Neuritis OR Polyradiculoneuropathy OR Guillain-Barre OR Miller Fisher OR Stiff-Person OR Uveomeningoencephalitic Syndrome OR Vogt Koyanagi Harada disease OR Vasculitis OR Arteritis OR Myositis OR Lichenoid Eruptions OR Lichen OR Pityriasis Lichenoides OR Psoriasis OR Psoriases OR Pustulosis OR Psoriatic OR Rheumatoid OR Wissler's OR Rheumatic OR Scleroderma OR CREST Syndrome OR Colitis OR Crohn OR Crohns OR Ankylosing Spondylitis OR Sialadenitis OR Parotitis OR Thyroiditis OR Hashimoto Disease OR Panniculitis OR Sarcoidosis OR Uveoparotid Fever OR Enteritis OR Ileitis OR Myocarditis OR Postpericardiotomy Syndrome OR Endocarditis OR Cystitis OR Liver Cirrhosis OR Sclerosing Cholangitis OR antisynthetase syndrome OR Alopecia Areata OR titis OR Hives OR Asthma OR Asthmas OR Epidermolysis Bullosa Acquisita OR Erythema Nodosum OR Hidradenitis Suppurativa OR Vitiligo OR oophoritis OR Endometriosis OR Orchitis OR enteropathy OR enteropathies OR Celiac Disease OR Coeliac disease OR sprue OR Evans Syndrome OR Cryoglobulinemia OR Red-Cell Aplasia OR Thrombocytopenia OR Adiposis Dolorosa OR Fasciitis OR Mikulicz Disease OR Orbital Pseudotumor OR Sinusitis OR Tuberculosis OR Plasma Cell Granuloma, Pulmonary OR Fibrosis OR Nephritis OR

	Angiolymphoid Hyperplasia with Eosinophilia OR Mixed Connective Tissue Disease OR rheumatism OR Facial Hemiatrophy OR Neuritis OR Schnitzler Syndrome OR Fibromyalgia OR Isaacs Syndrome OR Paraneoplastic Cerebellar Degeneration OR Hashimoto's encephalitis OR Restless Legs Syndrome OR Sydenham OR Uveitis OR Cogan Syndrome OR Plasminogen Deficiency, Type I OR Opsoclonus Myoclonus OR Scleritis OR Susac Syndrome OR Tolosa Hunt Syndrome OR Behcet Syndrome OR Meniere Disease OR Mucocutaneous Node Syndrome OR Polymyalgia Rheumatica OR Vasculitis OR Postmyocardial infarction syndrome OR Ords thyroiditis OR neutropenia OR Eosinophilic fascilitis OR IgG4 related disease OR Acute motor axonal neuropathy OR Moorens ulcer OR inner ear disease OR pancreatitis OR Graft vs Host Diseas OR Host vs Graft Reaction OR Rejection OR Rejections OR Graft vs Host Reaction OR Diseases, Graft Versus Host OR Disease, runt OR runting disease OR runting syndrome OR GVH disease OR allogeneic disease OR transplantation reaction OR Disease, Graft vs Host OR Homologous Wasting Disease OR Disease, Homologous Wasting OR antiphospholipid syndrome OR immunoglobulin A nephropathy OR IgA nephropathy OR IPEX syndrome OR immunoglobulin G4 related disease OR mobus crohn OR regional enterocolitis OR cleron disease OR Beauvais Disease OR athrosynovitis OR arthrochondritis OR chariot disease OR psorasiasiform OR willan lepra OR enthesitis OR Blau syndrome OR eczema OR allergy OR Bursitis OR Synovitis OR radiculitis OR Gastritis OR Esophagitis OR Inflammation OR inflammatory).ti,ab.
Animal studies	exp animal experiment/ or exp animal model/ or exp experimental animal/ or exp transgenic animal/ or exp male animal/ or exp female animal/ or exp juvenile animal/ OR animal/ OR chordata/ OR vertebrate/ OR tetrapod/ OR exp fish/ OR amniote/ OR exp amphibia/ OR mammal/ OR exp reptile/ OR exp sauropsid/ OR therian/OR exp monotremate/ OR placental mammals/ OR exp marsupial/ OR Euarchontoglires/ OR exp Afrotheria/ OR exp Boreoeutheria/ OR exp Laurasiatheria/ OR exp Xenarthra/ OR primate/ OR exp Dermoptera/ OR exp Glires/ OR exp Scandentia/ OR Haplorhini/ OR exp prosimian/ OR simian/ OR exp tarsiiform/ OR Catarrhini/ OR exp Platyrrhini/ OR ape/ OR exp Cercopithecidae/ OR hominid/ OR exp hylobatidae/ OR exp chimpanzee/ OR exp gorilla/ OR exp orang utan/ OR (animal OR animals OR pisces OR fish OR fishes OR catfish OR catfishes OR sheatfish OR silurus OR arius OR heteropneustes OR clarias OR gariepinus OR fathead minnow OR fathead minnows OR pimephales OR promelas OR cichlidae OR trout OR trouts OR char OR chars OR salvelinus OR salmo OR oncorhynchus OR guppy OR guppies OR millionfish OR poecilia OR goldfish OR goldfishes OR carassius OR auratus OR mullet OR mullets OR mugil OR curema OR shark OR sharks OR cod OR cods OR gadus OR morhua OR carp OR carps OR cyprinus OR carpio OR killifish OR eel OR eels OR anguilla OR zander OR sander OR lucioperca OR stizostedion OR turbot OR turbots OR psetta OR flatfish OR flatfishes OR plaice OR pleuronectes OR platessa OR tilapia OR tilapias OR oreochromis OR sarotherodon OR common sole OR dover sole OR solea OR zebrafish OR zebrafishes OR danio OR rerio OR seabass OR dicentrarchus OR labrax OR morone OR lamprey OR lampreys OR petromyzon OR pumpkinseed OR pumpkinseeds OR lepomis OR gibbosus OR herring OR clupea OR harengus OR amphibia OR amphibian OR amphibians OR anura OR salientia OR frog OR frogs OR rana OR toad OR toads OR bufo OR xenopus OR laevis OR bombina OR epidalea OR calamita OR salamander OR salamanders OR newt OR newts OR triturus OR reptilia OR reptile OR reptiles OR bearded dragon OR pogona OR vitticeps OR iguana OR iguanas OR lizard OR lizards OR anguis fragilis OR turtle OR turtles OR snakes OR snake OR aves OR bird OR birds OR quail OR quails OR coturnix OR bobwhite OR colinus OR virginianus OR poultry OR poultries OR fowl OR fowls OR

	chicken OR chickens OR gallus OR zebra finch OR taeniopygia OR guttata OR canary OR canaries OR serinus OR canaria OR parakeet OR parakeets OR grasskeet OR parrot OR parrots OR psittacine OR psittacines OR shelduck OR tadorna OR goose OR geese OR branta OR leucopsis OR woodlark OR lullula OR flycatcher OR ficedula OR hypoleuca OR dove OR doves OR geopelia OR cuneata OR duck OR ducks OR greylag OR graylag OR anser OR harrier OR circus pygargus OR red knot OR great knot OR calidris OR canutus OR godwit OR limosa OR lapponica OR meleagris OR gallopavo OR jackdaw OR corvus OR monedula OR ruff OR philomachus OR pugnax OR lapwing OR peewit OR plover OR vanellus OR swan OR cygnus OR columbianus OR bewickii OR gull OR chroicocephalus OR ridibundus OR albifrons OR great tit OR parus OR aythya OR fuligula OR streptopelia OR risoria OR spoonbill OR platalea OR leucorodia OR blackbird OR turdus OR merula OR blue tit OR cyanistes OR pigeon OR pigeons OR columba OR pintail OR anas OR starling OR sturnus OR owl OR athene noctua OR pochard OR ferina OR cockatiel OR nymphicus OR hollandicus OR skylark OR alauda OR tern OR sterna OR teal OR crecca OR oystercatcher OR haematopus OR ostralegus OR shrew OR shrews OR sorex OR araneus OR crocidura OR russula OR european mole OR talpa OR chiroptera OR bat OR bats OR eptesicus OR serotinus OR myotis OR dasycneme OR daubentonii OR pipistrelle OR pipistrellus OR cat OR cats OR felis OR catus OR feline OR dog OR dogs OR canis OR canine OR canines OR otter OR otters OR lutra OR badger OR badgers OR meles OR fitchew OR fitch OR foumart or foulmart OR ferrets OR ferret OR polecat OR polecats OR mustela OR putorius OR weasel OR weasels OR fox OR foxes OR vulpes OR common seal OR phoca OR vitulina OR grey seal OR halichoerus OR horse OR horses OR equus OR equine OR equidae OR donkey OR donkeys OR mule OR mules OR pig OR pigs OR swine OR swines OR hog OR hogs OR boar OR boars OR porcine OR piglet OR piglets OR sus OR scrofa OR llama OR llamas OR lama OR glama OR deer OR deers OR cervus OR elaphus OR cow OR cows OR bos taurus OR bos indicus OR bovine OR bull OR bulls OR cattle OR bison OR bisons OR sheep OR sheeps OR ovis aries OR ovine OR lamb OR lambs OR mouflon OR mouflons OR goat OR goats OR capra OR caprine OR chamois OR rupicapra OR leporidae OR lagomorpha OR lagomorph OR rabbit OR rabbits OR oryctolagus OR cuniculus OR laprine OR hares OR lepus OR rodentia OR rodent OR rodents OR murinae OR mouse OR mice OR mus OR musculus OR murine OR woodmouse OR apodemus OR rat OR rats OR rattus OR norvegicus OR guinea pig OR guinea pigs OR cavia OR porcellus OR hamster OR hamsters OR mesocricetus OR cricetulus OR cricetus OR gerbil OR gerbils OR jird OR jirds OR meriones OR unguiculatus OR jerboa OR jerboas OR jaculus OR chinchilla OR chinchillas OR beaver OR beavers OR castor fiber OR castor canadensis OR sciuridae OR squirrel OR squirrels OR sciurus OR chipmunk OR chipmunks OR marmot OR marmots OR marmota OR suslik OR susliks OR spermophilus OR cynomys OR cottonrat OR cottonrats OR sigmodon OR vole OR voles OR microtus OR myodes OR glareolus OR primate OR primates OR prosimian OR prosimians OR lemur OR lemurs OR lemuridae OR loris OR bush baby OR bush babies OR bushbaby OR bushbabies OR galago OR galagos OR anthropoidea OR anthropoids OR simian OR simians OR monkey OR monkeys OR marmoset OR marmosets OR callithrix OR cebuella OR tamarin OR tamarins OR saguinus OR leontopithecus OR squirrel monkey OR squirrel monkeys OR saimiri OR night monkey OR night monkeys OR owl monkey OR owl monkeys OR douroucoulis OR aotus OR spider monkey OR spider monkeys OR ateles OR baboon OR baboons OR

	papio OR rhesus monkey OR macaque OR macaca OR mulatta OR cynomolgus OR fascicularis OR green monkey OR green monkeys OR chlorocebus OR vervet OR vervets OR pygerythrus OR hominoidea OR ape OR apes OR hylobatidae OR gibbon OR gibbons OR siamang OR siamangs OR nomascus OR symphalangus OR hominidae OR orangutan OR orangutans OR pongo OR chimpanzee OR chimpanzees OR pan troglodytes OR bonobo OR bonobos OR pan paniscus OR gorilla OR gorillas OR troglodytes).ti,ab.
