# Supplementary material for: Immune responses to azacytidine in animal models of inflammatory disorders: a systematic review
Source: J Transl Med. 2021 Jan 6;19:11. doi: 10.1186/s12967-020-02615-2 (PMC7788785; doi:10.1186/s12967-020-02615-2)
Supplement: Supplementary file 2 — Additional file 2: Figure S1. Risk of Bias assessment and reporting of quality indicators of the included studies per study. [file 12967_2020_2615_MOESM2_ESM.pdf]

	Risk of Bias								Quality factors					
	1	2	3	4	5	6	7	8	1	2	3	4	5	6
Paluska Cihak 1982 Immunobiology	?	y	?	?	?	?	?	y	n	n	n	n	n	n
Sula, Cihak, 1987, Czech Med	?	?	?	?	?	?	?	?	n	n	n	n	n	n
Sánchez-Aberca, Pérez-simon, 2010, the american society of hematology	?	?	?	?	?	?	?	y	n	n	n	n	y	y
Choi, Dipersio, 2010, the american society of hematology	?	?	?	?	?	?	?	y	n	n	n	n	y	y
Fransolet, Baron, 2016, Journal of hematology and oncology	?	?	?	?	n	?	y	y	y	n	n	y	y	y
Cooper, DiPersio, 2017, Journal of immunology	?	?	?	?	?	?	?	?	y	n	n	n	y	y
Ehx,Baron 2017 OncoImmunology	?	?	?	?	?	?	?	?	n	n	n	n	y	y
Cheng C, Xia J, 2014, Immunology	?	?	?	?	?	?	?	n	n	n	n	n	y	y
Guo, Jiang, 2013, Transplant immunology	?	?	?	?	?	?	?	y	n	n	n	n	y	n
Wang X, Tao, 2017, Oncotarget	?	y	?	?	?	?	?	?	n	n	n	n	y	y
Uchida, Tisdale, 2014, PLOS ONE	?	?	?	?	?	?	?	?	n	n	n	n	y	n
Hong J, Yang, 2013, Transplant immunology	?	?	?	?	?	?	?	y	n	n	n	n	y	y
Zheng, Zhao, 2009, J mol med	?	y	?	?	?	?	?	y	n	n	n	y	y	y
Wang, Shi, 2016, JCI insight	?	?	?	?	?	?	?	?	n	n	n	y	y	n
Gao, Mu, 2019, Stem Cell Research and Therapy	?	?	?	?	?	?	?	y	n	y	n	y	y	y
Chen, Dong, 2019, Kidney international	?	?	?	?	?	?	?	?	n	y	n	y	y	y
Zhang, Liang, 2017, Kidney International	?	y	?	?	?	?	?	y	n	y	n	n	y	y
Cao, Xua , 2014, Endocrinology	?	?	?	?	?	?	?	?	n	y	n	n	y	y
Li, Tang, 2016, the american physiological society	?	y	?	?	?	?	?	?	n	n	n	n	y	y
Yoshida, Izni, 1990, European journal of immunology	?	?	?	?	?	?	?	?	n	n	n	n	n	n
Mizugaki, Nose, 1997, Clin Exp Immunol	?	?	?	?	?	?	?	?	n	n	n	n	n	n
Li, Tsokos, 2018, JCI Insight	?	?	?	?	?	?	?	?	n	n	n	n	y	y
Sreekumar, Smyth, 1996, Clin immunol immunopathol	?	?	?	?	?	?	?	?	n	n	n	n	n	n
Huang, Wang, 2016, Biomedicine & Pharmacotherapy	?	?	?	?	?	?	?	y	n	y	n	y	y	y
Cui, Shang, 2019, Laboratory Investigation	?	y	?	?	?	?	y	?	y	y	n	y	y	y
Singer, D'alessio, 2014, Am Journal of Respiratory Cell and Molecular Biology	?	?	?	?	?	?	?	y	n	n	y	n	y	y
Thangavel, Rajasingh, 2014, Am Journal of Pathology	?	?	?	?	?	?	y	?	n	y	n	n	y	n
Thangavel, Rajasingh, 2015, Journal of Cell Science	?	?	?	?	?	?	?	?	n	n	n	n	y	y
Wu, Kuo, 2012, Allergy and Immunology	?	?	?	?	?	?	?	?	n	n	n	n	y	n
Brand, Renz, 2011, Journal of Allergy and Clinical Immunology	?	?	?	?	?	?	y	y	y	n	n	n	n	y
Kröger, Ehrlich, 1999, General Pharmacology	?	?	?	?	?	?	?	y	n	n	n	n	n	n
Tóth, Rauch, 2019, Arthritis & Rheumatology	?	y	?	?	?	?	?	?	y	n	n	n	y	y
Chan, Wu, 2014, Molecular medicine	?	?	?	?	?	?	?	?	n	n	n	y	y	y
Mangano, Nicoletti, 2014, Journal of Cellular Physiology	?	y	?	?	?	?	?	y	n	n	n	y	y	y
Fagone, Nicoletti, 2018, Journal of Neuroimmunology	?	y	?	?	?	?	?	?	y	y	n	y	y	y

Questions

Risk of Bias

1. Was the allocation sequence adequately gereated and applied?
2. Were the groups similar at baseline or adjusted for confounders?
3. Was the allocation adequately concealed?
4. Are the animals randomly housed during the experiment?
5. Were the caregivers/investigators during the course of the exp adequately blinded?
6. Were animals selected at random during outcome assessment?
7.Was the outcome assessment adequately blinded?
8. Were incomplete outcome data adequately addressed?

Quality factors:

1. Is any blinding reported?
2. Is any randomisation reported?
3. Is the sample size calculation reported?
4.Are housing conditions reported?
5. Is ethical approval reported?
6. Are any conflicts of interest reported?
